# Exploring the effect of gut flora on atrial fibrillation based on epidemiological history

**DOI:** 10.3389/fcvm.2025.1655007

**Published:** 2025-12-19

**Authors:** Chunxiao Wang, Mengyi Shen, Yueyu Zhao, Zili Xu, Yuxin Huang, Hongjie Xiang

**Affiliations:** 1 College of Traditional Chinese Medicine, Shandong Second Medical University, Weifang, China; 2 The First Affiliated Hospital of Shandong First Medical University & Shandong Provincial Qianfoshan Hospital, Jinan, China; 3 Shandong University of Chinese Medicine, Jinan, China; 4 Malan Township Health Centre, Taierzhuang District, Zaozhuang, China; 5 Jiangguantun Health Community Service Centre, Liaocheng, China; 6 Department of Traditional Chinese Medicine, The First Affiliated Hospital of Shandong First Medical University & Shandong Provincial Qianfoshan Hospital, Jinan, China

**Keywords:** atrial fibrillation, autonomic nervous system, gut flora, inflammasome, microbial metabolites, myocardial fibrosis

## Abstract

Emerging evidence from preclinical and observational studies indicates an association between gut flora dysbiosis and atrial fibrillation (AF). The gut flora constitutes a highly complex and dynamic community capable of producing diverse metabolites that influence host pathophysiology. External factors, such as diet and medications, further modulate its composition. This review synthesizes current knowledge on the relationship between gut flora and AF, emphasizing the role of microbial-derived metabolites in the underlying mechanisms of AF. We also explore the involvement of gut flora in AF-related conditions, including hypertension and diabetes. Additionally, we evaluate potential interventional strategies targeting the gut flora through dietary or pharmacological approaches for their antiarrhythmic effects. Finally, we address limitations in the current evidence and propose future research directions to clarify the causal relationship and therapeutic potential of targeting the gut flora in AF. While this review synthesizes the current evidence linking gut flora to AF, we also critically examine the limitations of the existing literature, including reliance on preclinical models, confounding factors in human studies, and inconsistencies in interventional outcomes, to provide a balanced perspective on the field's current state and future directions.

## Graphical Abstract

**Figure:**
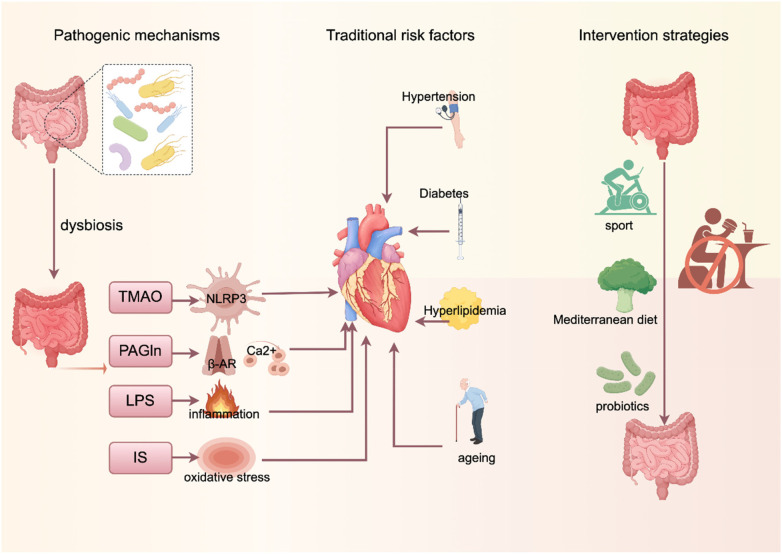
Created using Figdraw (https://www.figdraw.com/).

## 1 Introduction

Atrial fibrillation (AF) is an atrial disorder characterized by rapid and irregular beating (1), which results in the loss of effective atrial pumping function and imposes a significant health burden on patients and society. Although the prevention and treatment of AF and its associated risk factors have improved considerably in recent years, the incidence of AF continues to rise, affecting more than 37 million people worldwide (2).

Both clinical and preclinical studies have shown that the onset and progression of AF are significantly influenced by a variety of modifiable cardiovascular risk factors, including hypertension (3), diabetes mellitus (4) and obesity (5). It is worth noting that most of these risk factors are closely related to dietary structure and lifestyle (6). After food is digested and enters the gut, it interacts with the gut flora, a large and complex symbiotic ecosystem. In recent years, abnormal alterations in gut microbial composition and function have been found to be potentially closely associated with a variety of cardiovascular-metabolic diseases (7). Dysbiosis can affect intestinal barrier function (8), promote inflammatory responses, and participate in host physiological and pathological regulation through its metabolites. However, whether and how the gut flora and its metabolites are directly involved in the development of AF remains unclear. The aim of this paper is to review the current evidence linking gut microbial profiles to AF, with a focus on the potential mechanisms of microbiota-derived metabolites in the pathogenesis of AF and their influence on cardiovascular disease-related risk factors. We also discuss the potential value of pharmacological and dietary interventions targeting the gut flora for the treatment of AF, and finally, identify knowledge gaps in the field and suggest directions for future research.

Finally, it is crucial to note that the current evidence predominantly stems from animal and observational studies. The establishment of a definitive causal relationship and the translation of these findings into effective therapies require validation through more robust clinical trials.

## 2 Dysregulation of metabolites of intestinal flora on the pathogenesis of AF

### 2.1 Trimethylamine oxide (TMAO)

TMAO, an important metabolite of gut flora, has been shown to be strongly associated with the development of AF (9). A study on diabetes-associated AF showed that (10) diabetic cardiomyopathy (DCM) model rats not only exhibited dysbiosis of the intestinal flora and elevated levels of systemic TMAO, but also had a significantly higher rate of AF induction than control subjects. Mechanistically, current research indicates that TMAO may promote the onset and progression of AF through several potential mechanisms, including inflammatory activation, promotion of thrombosis, and pro-atrial fibrosis. Krishna M. Boini et al. demonstrated in mice that TMAO activates the NOD-like receptor protein 3 (NLRP3) inflammasome (11). This activation, in turn, impairs tight junction proteins and endothelial cell permeability, leading to a cascade of events including reactive oxygen species (ROS) activation, lysosomal rupture, and potassium ion efflux, thereby increasing AF risk (11). But addition of a specific TMAO inhibitor to drinking water significantly reduces plasma TMAO levels (10) and attenuates inflammatory cell infiltration in atrial tissue, ameliorating atrial inflammation and connexin remodelling.

**Figure 1:**
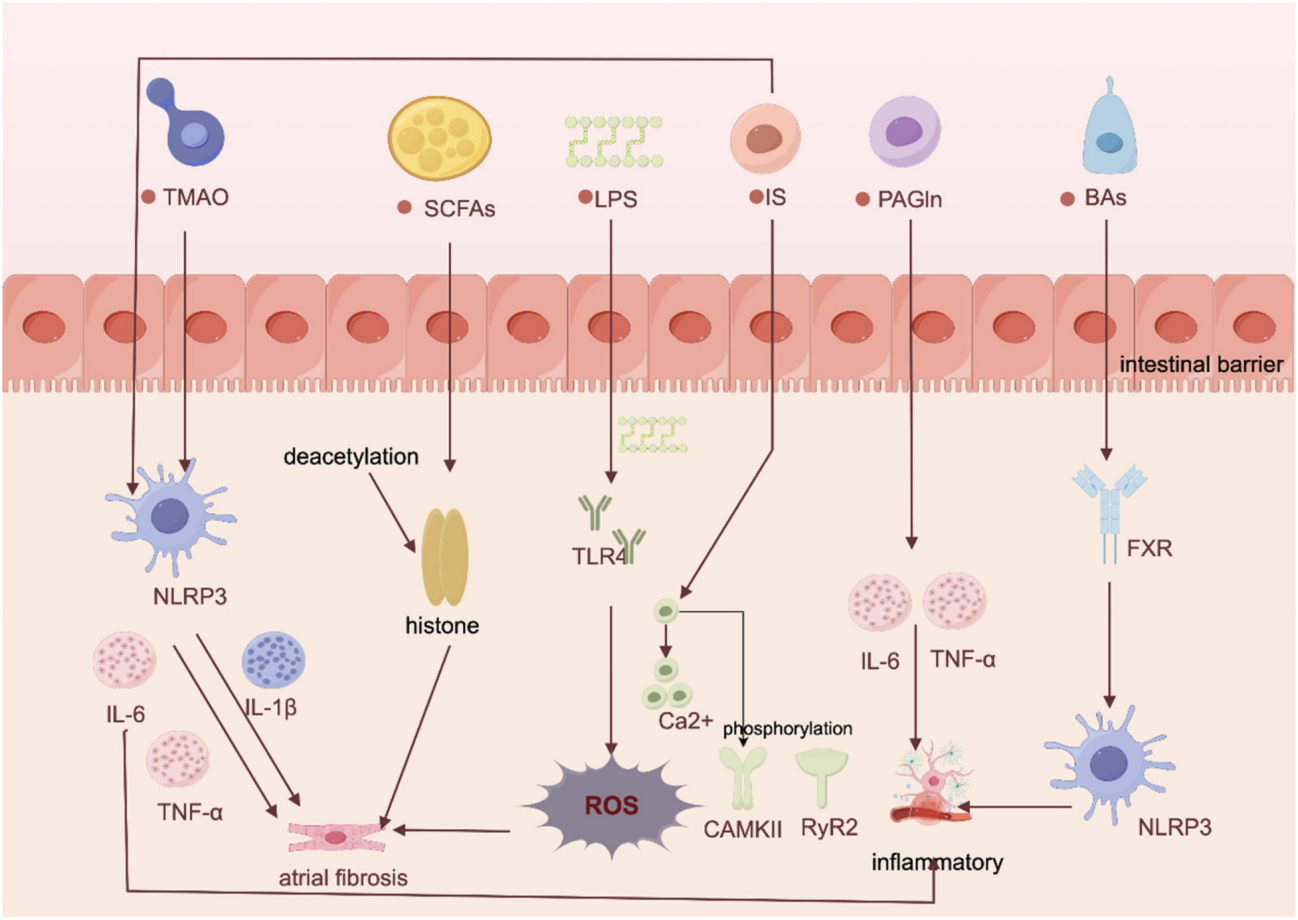
Proposed pathways and mechanisms linking gut flora-derived metabolites to AF development. Created using Figdraw (https://www.figdraw.com/).

TMAO can also increase the risk of thrombosis as it accelerates thrombus formation by inducing platelet aggregation, enhancing platelet reactivity, promoting tissue factor expression, and inducing endothelial cell injury (12, 13). The findings of Luo et al. that elevated plasma trimethylamine N-oxide enhances M1 macrophage infiltration in the atria, thereby inducing structural remodelling of the atria and increasing susceptibility to AF, provide further evidence for the mechanism by which TMAO induces AF (14). However, the translational relevance of these mechanistic pathways is challenged by clinical interventions targeting TMAO production. Specifically, randomized controlled trials demonstrate that supplementation with lactobacilli, a common probiotic, fails to significantly reduce circulating TMAO levels in humans (15, 16). Therefore, TMAO levels represent a promising candidate biomarker for predicting AF risk. However, its clinical utility requires further validation in diverse human cohorts, independent of renal function and other cardiovascular comorbidities (Figure 1).

### 2.2 Endotoxins (lipopolysaccharide, LPS)

LPS is a major component of the cell membrane of gram-negative bacteria, and when the intestinal barrier function is impaired, LPS can enter the circulation and promote chronic systemic inflammation by activating a strong immune response (17). It has been suggested that chronic high-fat diets create favorable conditions for the proliferation of gram-negative bacteria (18), and is one of the causes of impaired intestinal barrier function (14). In terms of mechanisms (14), the high-fat diet tends to impair the intestinal barrier, leading to elevated circulating levels of LPS and the development of a pro-inflammatory state, for reasons related to the elevation of macrophage markers of inflammation signalled by LPS via Toll-like receptor 4 (TLR4) and CD14, but all of the above are reversible after antibiotic treatment. In addition, Zhang et al. demonstrated that (19) aging-associated gut flora dysbiosis enhances atrial NLRP3 inflammasome activity by increasing circulating LPS and glucose levels. This process promotes atrial fibrosis and ultimately contributes to AF development, providing compelling evidence for LPS's direct role in AF pathogenesis. Furthermore, the NLRP3 inflammatory signaling pathway was significantly enhanced in cardiomyocytes from patients with paroxysmal, persistent and postoperative AF (20). Upregulation of the NLRP3 system not only leads to atrial fibrosis but may also trigger arrhythmias by shortening the atrial action potential and increasing the frequency of Ca²⁺ release from the spontaneous diastolic sarcoplasmic reticulum, inducing delayed afterdepolarization and ectopic activity (21).

### 2.3 Short-chain fatty acids (SCFAs)

SCFAs are the main metabolites of fermentation of dietary fibre and glucose by colonic microbiota, and mainly include acetate, propionate and butyrate (22). SCFAs enhance intestinal barrier function, inhibit histone deacetylases (23), modulate immune responses, and regulate metabolic and inflammatory responses through G protein-coupled receptors (GPCRs). They are also involved in cardiac repair and arterial compliance regulation after ischemia/reperfusion injuries and myocardial infarction (24). However, there are differences in the role of different SCFAs in cardiovascular disease. Butyrate exerts beneficial metabolic effects through its anti-inflammatory action and increased insulin sensitivity (25). Propionate, on the other hand, reduces the risk of obesity by stimulating the release of glucagon-like peptide-1 (GLP-1) and peptide YY (PYY) and affects blood pressure by modulating renin release (26). Whereas acetate may promote insulin and growth hormone-releasing peptide secretion through activation of the parasympathetic nervous system, leading to dyslipidaemia and obesity (27).

However, that the evidence linking specific probiotic bacteria, which are putative enhancers of SCFA production, to these benefits is not always robust. For instance, meta-analyses on Bifidobacterium abundance indicate that the overall significance often hinges on a few studies, as the pooled result loses statistical significance upon the exclusion of specific trials (28–30). This highlights the fragility and inconsistency of the current evidence base connecting probiotic interventions to the SCFAs-related mechanisms discussed above (Table 1).

**Table 1:** Dysregulated Gut Flora-derived metabolites and their pathogenic roles in atrial fibrillation.

Metabolite class	Representative molecules	Key pathophysiological mechanisms	Impact on AF
TMAO	Trimethylamine N-oxide	1. Activation of NLRP3 inflammatory vesicles promotes atrial inflammation.	Increased susceptibility and persistent risk of atrial fibrillation; biomarkers for predicting the occurrence of atrial fibrillation.
		2. Enhances platelet reactivity and promotes thrombosis.	
		3. Induces M1 macrophage infiltration and promotes structural remodelling of atrial fibrosis.	
LPS	Lipopolysaccharide	1. Systemic chronic inflammation via the TLR4/CD14 pathway.	Disrupts electrophysiological stability, promotes atrial fibrosis and increases the risk of atrial fibrillation.
		2. Enhancement of atrial NLRP3 inflammatory vesicle activity.	
		3. Leads to oxidative stress and abnormal calcium handling.	
PAGln	Phenylacetylglutamine	1. Agonises adrenergic *β*2 receptors (β2-AR), enhancing platelet activity and calcium cycling.	Is an independent predictor of atrial fibrillation in patients with heart failure and renal disease.
		2. Increased ROS production and leading to calcium leakage and posterior depolarisation.	
IS	Indoxyl sulfate	1. Induces oxidative stress and Ca²⁺ overload in cardiomyocytes.	It is strongly associated with the risk of atrial fibrillation associated primarily with chronic kidney disease.
		2. Promote the expression of inflammatory factors (TNF-α, IL-6, IL-1β).	
		3. It has a pro-fibrotic and pro-hypertrophic effect.	
SCFAs	Butyrate, acetate	A two-way street:	Butyrate may be protective; other SCFAs have complex roles and may indirectly influence AF risk factors.
		Beneficial (Butyrate based): Strengthens intestinal barrier, anti-inflammatory,	
		Harmful effects (acetate based): May promote obesity and dyslipidaemia via vagal stimulation.	
Bas	CDCA, UDCA	A two-way street:	CDCA promotes atrial remodelling; UDCA may have protective potential.
		Harmful (CDCA): activates FXR and NLRP3, promotes apoptosis and fibrosis; affects ion channels.	
		Beneficial (UDCA): Stabilises cell membrane potential and may be antiarrhythmic.	

NLRP3, NOD-like receptor thermoprotein structural domain-associated protein 3; TLR4, Toll-like receptor 4; ROS, activated oxygen; CaMKII, Calcium/calmodulin-dependent protein kinase II; RyR2, Ranibine receptor 2; FXR, Farnesol X receptor.

### 2.4 Phenylacetylglutamine (PAGln)

PAGln (31) is one of the metabolites of bacterial phenylalanine in humans, and is derived from the combination of phenylacetic acid and glutamine. A prospective clinical study confirms that elevated plasma PAGln promotes the expression of Interleukin-1β (IL-1β) and Interleukin-6 (IL-6) to increase the risk of developing AF (32). Moreover, PAGln is significantly elevated in end-stage renal disease, coronary artery disease (CAD) with stent stenosis (7), and has been used as an independent predictor of secondary AF in these diseases. Some animal studies have found that PAGln significantly increased the left atrial inner diameter (LAD) (33) and exacerbated the increase in plasma N-terminal pro-B-type natriuretic peptide (NT-proBNP) concentration as well as promoted the expression of atrial natriuretic peptide (ANP) and brain natriuretic peptide (BNP) mRNA in atrial tissue of heart failure (HF) mice. Mechanistically, by acting as an agonist of the adrenergic *β* receptor 2 (*β*2-AR) (7), PAGln can potentiate platelet reactivity/thrombosis in arterial injury models and enhance spontaneous calcium release linked to AF (34). Thus, *β*2 AR has been identified as a potential key mediator of PAGln's action on AF, though the *in vivo* significance in humans remains to be fully elucidated. In addition, PAGln induces arrhythmias by increasing the accumulation of ROS, a product of oxidative stress, to promote activation of calmodulin kinase II (CaMKII) and ryanodine receptor 2 (RyR2), and by increasing Ca2+ release and calcium overload (35) as a means of promoting excitability and afterdepolarization of the cell membrane.

### 2.5 Indolephenol sulphate (IS)

IS is a uremic toxin, produced by gut flora through dietary protein metabolism (36, 37).Currently confirmed (37), increased levels of IS in the body contribute to the development of AF by increasing oxidative stress to exacerbate inflammation and cardiac fibrosis. Owing to its high protein-binding capacity and poor dialyzability, serum levels of IS remain elevated even after hemodialysis (38). Consequently, serum IS is considered a biomarker for predicting cardiovascular events in patients with advanced chronic kidney disease (38). In mechanisms (39), IS can alter electrical activity and induce Ca2 + overload in cardiomyocytes through oxidative stress, which interrupts regulatory proteins of calcium channels and increases intracellular Ca2+ overload and Ca2+ release. They also demonstrated in cellular experiments that IS has a pro-inflammatory effect, leading to significant increases in the inflammatory cytokines tumour necrosis factor- *α*, IL-6 and IL-1β. Thus, IS may have pro-fibrotic, pro-hypertrophic and pro-inflammatory effects on cardiomyocytes.

### 2.6 Bile acids (BAs)

BAs are the end products of cholesterol metabolism, mainly including free bile acids, primary bound bile acids and secondary free bile acids (40). Among them, secondary free bile acids can act as hormone-like molecules and regulate signalling pathways such as metabolism, inflammation and energy expenditure (41). However, excessive free bile acids may be cardiotoxic. For example, chenodeoxycholic acid (CDCA) may promote AF by binding to amino acids to form bile salts, further stimulating cardiac sodium-calcium exchange, inducing changes in membrane potential, and activating muscarinic M2 receptor/acetylcholine-regulated potassium currents in cardiomyocytes (42). In addition, experimental data suggest that CDCA can promote cardiac injury and fibrosis via farnesol X receptor (FXR) activation in model systems and exacerbates the inflammatory response by activating NLRP3 inflammasome (43). Animal experiments further validated this mechanism, Wang (44) found that CDCA levels in patients with AFwere positively correlated with left atrial low-voltage areas and that CDCA showed a dose-dependent relationship with atrial myocyte apoptosis in a mouse model, suggesting an important role in structural remodelling in AF. On the other hand, certain bile acids such as ursodeoxycholic acid (UDCA) are cardioprotective, and UDCA, as a hydrophilic bile acid, may play an active role in the prevention of cardiac arrhythmias by stabilising the potential of cell membranes (45).

## 3 Interaction of gut flora with traditional cardiovascular risk factors

### 3.1 There is a bidirectional relationship between gut flora and hypertension

Hypertension is a major risk factors for many cardiovascular diseases (46), and its pathogenesis is closely linked to intestinal microbiota disorders. Studies suggest a bidirectional regulatory relationship exists between hypertension and gut flora (47, 48). Animal experiments have confirmed that significant intestinal pathological alterations occur in hypertensive rat models. These alterations, which include impaired intestinal barrier integrity, dysregulated immune responses, and hemodynamic changes, may contribute to the development and maintenance of hypertension (48). The main manifestations were increased plasma fluorescein isothiocyanate (FITC)-dextran concentration, decreased tight junction proteins, increased fibrotic area of the intestinal wall, thickening of the muscularis propria, decreased number of cuprocytes and shortening of intestinal cilia (49).

In contrast, the antihypertensive drug chlorosartan (50) restores the expression of colonic closure proteins and mucins by promoting *α*-defensin production and inhibiting sympathetic excitation (51), a finding that confirms the bi-directional regulatory relationship between hypertension and intestinal barrier function. Notably, studies have found significantly lower levels of butyrate in hypertensive patients (52, 53). Butyrate is highly active in regulating blood pressure, dilating blood vessels and reducing inflammatory responses (54). In terms of mechanisms (55), butyrate acts on the receptor GPR109A to control the development of hypertension by modulating the immune response so that macrophages exhibit a higher capacity to induce differentiation of juvenile cells to regulatory T cells. In addition, the acetate and propionate salts in SCFAs can also lower blood pressure by relaxing blood vessels (56, 57). Animal experiments confirmed that SCFAs significantly dilated mesenteric and thin femoral arteries in phenylephrine preconstricted rats (58). However, the regulation of blood pressure by SCFAs is bidirectional: when they bind to the vascular OLFR78 receptor they promote renin release, leading to an increase in blood pressure, whereas when they interact with the G-protein-coupled receptor GPR43 they exert a vasodilatory effect and reduce blood pressure (59). This receptor-dependent dual regulatory action reveals a complex mechanism of SCFAs in blood pressure regulation. Gut flora is also involved in the development of hypertension through immunomodulatory pathways. A high-salt diet can, in turn, induce the formation of a pro-inflammatory environment in the body, which in turn promotes the development of hypertension. Specific mechanisms involve T-cell activation/inflammatory cytokines/gut flora pathways, etc. (60). Notably, propionic acid in SCFAs has significant anti-inflammatory properties, inhibits the progression of cardiovascular disease in animal models, and exhibits antihypertensive effects (58), its cardioprotective effects are mainly dependent on regulatory T cells (Treg), which reduce cardiac hypertrophy, fibrosis, arrhythmia susceptibility and atherosclerotic lesions by regulating T helper cell homeostasis. Supplementation of SCFAs restores endotoxaemia and regulates Th17/Treg cell balance in mesenteric lymph nodes in hypertensive rats, thus exerting an antihypertensive effect (61).

In conclusion, intestinal microecology is closely related to the pathogenesis of hypertension. A dysregulated intestinal microenvironment is involved in traditional mechanisms of hypertension, such as the autonomic nervous system, renin-angiotensin-aldosterone system and the immune system, by altering the composition and distribution of the bacterial flora, influencing the function of the intestinal barrier, regulating the metabolites of the flora and mediating immune-inflammatory responses (62). These traditional mechanisms, in turn, interact with the gut flora and work together to promote the progression of hypertension (63). These findings provide an important theoretical basis for an in-depth understanding of the pathogenesis of hypertension and the development of new therapeutic strategies (Table 2).

**Table 2:** Summary of gut flora interactions with traditional cardiovascular risk factors.

Risk factor	Nature of interaction with Gut flora	Core mechanisms	Key microbial/metabolite changes	Key Insights and therapeutic implications
Hypertension	Bidirectional Regulation	1. Destruction of the intestinal barrier: Leads to endotoxemia and systemic inflammation.	Deleterious: Increased LPS, decreased butyrate	The gut flora is a central regulator of hypertension. Supplementing SCFAs, using specific probiotics, or drugs can lower blood pressure by repairing the intestinal barrier and modulating immunity.
		2. Immunomodulation: SCFAs regulate Treg cells via receptors, influencing blood pressure.	Beneficial: Propionate, acetate	
		3. Neurological & RAAS regulation: SCFAs bidirectionally regulate renin release via Olfactory Receptor 78 (OLFR78) and GPR43.		
		4. High-salt diet: Induces pro-inflammatory Th17 cell response and disrupts microbial balance.		
Insulin Resistance & Diabetes	Driver	1. Inflammation: Microbiota-derived LPS triggers chronic low-grade inflammation via the TLR4 pathway, interfering with insulin signaling.	Deleterious: Increased LPS, BCAAs, succinate Beneficial: Decreased SCFAs, indole derivatives	The gut flora is central to the “chronic inflammation” of T2DM. Targeting the microbiota can reduce LPS translocation and increase beneficial metabolites, offering new strategies for preventing diabetes and associated AF.
		2. Metabolite regulation: SCFAs, indole derivatives exert protective effects by stimulating GLP-1 secretion and improving insulin sensitivity; BCAAs and succinate promote resistance.		
		3. Iron overload: Gut flora-associated iron dysregulation reduces insulin secretion and promotes resistance.		
Hyperlipidemia	Main Regulator	1. Lipid metabolism regulation: Microbiota modulates lipid balance by affecting epithelial integrity, cholesterol metabolism, and lipid oxidation/storage.	Deleterious: Increased Firmicutes, TMAO	Blood lipid levels are closely tied to microbiota composition. Reducing TMAO levels and supplementing SCFAs are novel approaches to modulate lipids and reduce cardiovascular risk.
		2. TMAO pathway: High-fat/high-choline diet → promotes gut microbial TMA production → hepatic FMO3 oxidation to TMAO → promotes atherosclerosis and thrombosis.	Beneficial: Butyrate, propionate	
		3. SCFAs effects: Butyrate and propionate lower cholesterol and triglycerides by activating receptors		
Aging	Accelerator	1. Microbial evolution: With age, glycolytic bacteria decrease, while proteolytic bacteria increase.	Protective decrease: Butyrate-producing bacteria	Age-related dysbiosis is an independent risk factor for AF. Transplanting aged microbiota increases AF susceptibility. Modulating the microbiota during aging (e.g., increasing dietary fiber) may delay cardiovascular aging and prevent age-related AF.
		2. Inflammation & aging: Increased entry of harmful metabolites into circulation activates the NLRP3 inflammasome, promoting atrial fibrosis.	Deleterious increase: Proteolytic bacteria, PAGln, TMAO	
		3. Molecular aging: PAGln induces mitochondrial dysfunction and DNA damage via the ADR-AMPK pathway; telomere dysfunction exacerbates gut leakiness and inflammation		

SCFAs, short-chain fatty acids; TMAO, trimethylamine N-oxide; LPS, lipopolysaccharide; Treg, regulatory T cells; RAAS, renin-angiotensin-aldosterone system; PAGln, phenylacetylglutamine; BCAAs, branched-chain amino acids.

### 3.2 Dysbiosis of gut flora is associated with insulin resistance

Diabetes mellitus (DM) is an established independent risk factor for AF. And the gut flora has been suggested as a therapeutic target for pre-diabetes and early diagnosis (64),that can lead to diabetes by affecting intestinal permeability, inflammation, the immune system and energy metabolism.

One study (65) revealed the central role of the inflammatory response in the development of type 2 diabetes mellitus (T2DM) (66). T2DM is now redefined as a chronic inflammatory disease (10). Insulin resistance (IR) is strongly associated with a chronic low-grade inflammatory state (67), this chronic inflammation interferes with insulin signalling and reduces insulin bioavailability through direct or indirect mechanisms. Notably, endotoxaemia induced by LPS produced by gut flora may be a key trigger of inflammation and insulin resistance. After bacterial lysis, LPS enters the circulation and triggers systemic chronic low-grade inflammation (67) through activation of Toll-like receptor 4 (TLR4), which in turn induces serine phosphorylation of insulin receptor substrate-1, ultimately leading to the development of insulin resistance.

Mechanistically, SCFAs are produced in the intestinal tract by the phylum Serratia marcescens and Serratia marcescens to stimulate the release of glucagon-like peptide-1 (GLP-1) and activate the G protein-coupled receptor (gpcr) expressed in enteroendocrine cells, inducing insulin secretion from the pancreas (65). In addition, indole and its derivatives have been shown to bind and activate the aromatic hydrocarbon receptor (AhR), which is associated with improved insulin secretion and insulin sensitivity. N-acylamides have also been found to enhance glucose metabolism in humans by binding to G protein-coupled receptor 119 (GPR119). However, branched chain amino acids (BCAAs) and succinate from bacteria can accelerate the development of insulin resistance (68). In addition gut flora-associated iron overload increases susceptibility to type 2 diabetes by reducing insulin secretion, promoting insulin resistance and facilitating hepatic gluconeogenesis (69).

DM is strongly associated with elevated levels of systemic inflammatory mediators (70), study shows significant activation of multiple pro-inflammatory pathways in myocardial tissue of diabetes model animals (71),suggests chronic cardiac inflammation may be an important pathophysiological bridge between diabetes and arrhythmias (66). Specifically, a persistent low-grade inflammatory state may lead to altered myocardial electrophysiological properties, increased fibrosis, and autonomic dysfunction, which may increase the risk of arrhythmias such as AF. This mechanism provides a new perspective for understanding the pathogenesis of diabetes-associated AF, as well as a theoretical basis for the development of therapeutic approaches based on anti-inflammatory strategies.

### 3.3 Intestinal flora and hyperlipidaemia

Significant correlation between blood lipid levels and cardiovascular disease incidence (72). The relative abundance of Firmicutes was significantly higher in patients with hyperlipidemia, a finding that suggests a strong association between the composition of the intestinal flora and blood lipid levels (73). Gut flora are involved in the regulation of lipid metabolism by altering the integrity of intestinal epithelial cells, regulating cholesterol metabolism, promoting lipid oxidation in muscle tissue, and modulating lipid storage in adipose tissue (74), these mechanisms work together to maintain the body's lipid metabolic balance. Recent studies have revealed the key role of gut flora metabolites in lipid metabolism. Butyrate in SCFAs exerts a lipid-regulating effect by activating the intestinal GPR109A receptor, which in turn activates macrophages, reduces total lipase activity and inhibits triglyceride synthesis and accumulation (26). In addition, *in vitro* and *in vivo* studies have demonstrated that propionic acid reduces cholesterol levels by activating free fatty acid receptors GPR41 and GPR43, which promote the secretion of glucagon-like peptide-1 (GLP-1) and peptide YY (PYY) (75). It is worth noting that TMAO is strongly associated with high-fat diets. These diets are rich in choline, a key substrate for TMAO production. Choline is first broken down in the gut to trimethylamine (TMA), which is subsequently oxidized by hepatic flavin monooxygenase 3 (FMO3) to produce TMAO (76). It was further found that a high-fat diet alters intestinal epithelial physiology by impairing colonic epithelial mitochondrial bioenergetics and eliminating epithelial hypoxia, promoting intestinal TMA production and increasing circulating TMAO levels (77). A randomised crossover design study confirms that a diet high in saturated fat increases systemic TMAO levels by increasing dietary precursors and reducing renal TMAO excretion (17),this finding consistent with clinical observations of elevated plasma TMAO levels.

Based on the available evidence, the intestinal flora in hyperlipidemic patients increases the risk of AF mainly by affecting lipid metabolism, altering intestinal epithelial integrity and permeability, and increasing the level of TMAO *in vivo*, these mechanisms may ultimately increase the risk of AF by promoting atrial remodelling, increasing inflammatory responses and oxidative stress. These findings provide new perspectives for understanding the pathogenesis of hyperlipidaemia-associated AF and a theoretical basis for developing intervention strategies based on the regulation of gut flora.

### 3.4 Correlation of ageing gut flora with age

The risk of developing AF was significantly and positively associated with age, a phenomenon that may be closely related to age-related changes in gut flora. Research suggests that the gut flora plays a key role in regulating the organism's aging process (78), and the dynamics of gut flora with age is an independent risk factor for the development of atrial fibrillation (79).

With age, the composition of the gut flora shows a decrease in the proportion of glycolytic bacteria and an increase in the proportion of proteolytic bacteria (80). Glycolytic bacteria provide cardiovascular protection by producing beneficial metabolites such as short-chain fatty acids (SCFA) (for example, butyrate),which can maintain the intestinal barrier function (81, 82) and reduces the entry of pro-inflammatory molecules such as LPS, TMAO and PAGln into the blood circulation by, thus regulating macrophage activity and inhibiting atherosclerotic plaque formation. On the other hand, proteolytic bacteria may produce potentially harmful metabolites such as trimethylamine, which is converted to trimethylamine oxide (TMAO) in the liver (83), which may accelerate the ageing process of endothelial cells and the vascular system by activating oxidative stress pathways.

Its animal experiments further confirmed this mechanism, as transplantation of faecal flora from aged rats (22–24 months old) to young rats (2–3 months old) significantly increased atrial NLRP3 inflammasome activity, exacerbated atrial fibrosis and increased AF susceptibility, whereas reverse transplantation showed a protective effect. Other evidence for this conclusion comes from Zhang et al's (19) research which reveals that gut dysbiosis associated with aging promotes AF through multiple mechanisms: including increased circulating LPS and glucose levels, enhanced atrial NLRP3 inflammasome activity, and ultimately atrial fibrosis.

At the microscopic level, telomere wear, epigenetic alterations, loss of protein homeostasis, mitochondrial dysfunction and altered intercellular communication have been recognised as major molecular and cellular hallmarks of ageing (78, 84). These biological aging, in turn, are directly related to the decline in the functioning of the flora. Yang et al. found that (85) increased production of PAGln in the gut of the elderly and validation of the PAGln -induced senescence phenotype in cellular and mouse models suggests a mechanism by which PAGln induces mitochondrial dysfunction and DNA damage via the adrenergic receptor (ADR)-amp activated protein kinase (AMPK) signalling pathway. And telomere dysfunction may also directly affect the integrity of intestinal tissue (86),this leads to increased leaky gut, systemic inflammation and ecological dysregulation, and further accelerates telomere shortening. For example, telomerase-deficient mice have shorter intestinal telomeres than wild-type, reduced expression of intestinal genes that tightly link the F11 receptor, and increased expression of inflammatory markers (87).

**Figure 2:**
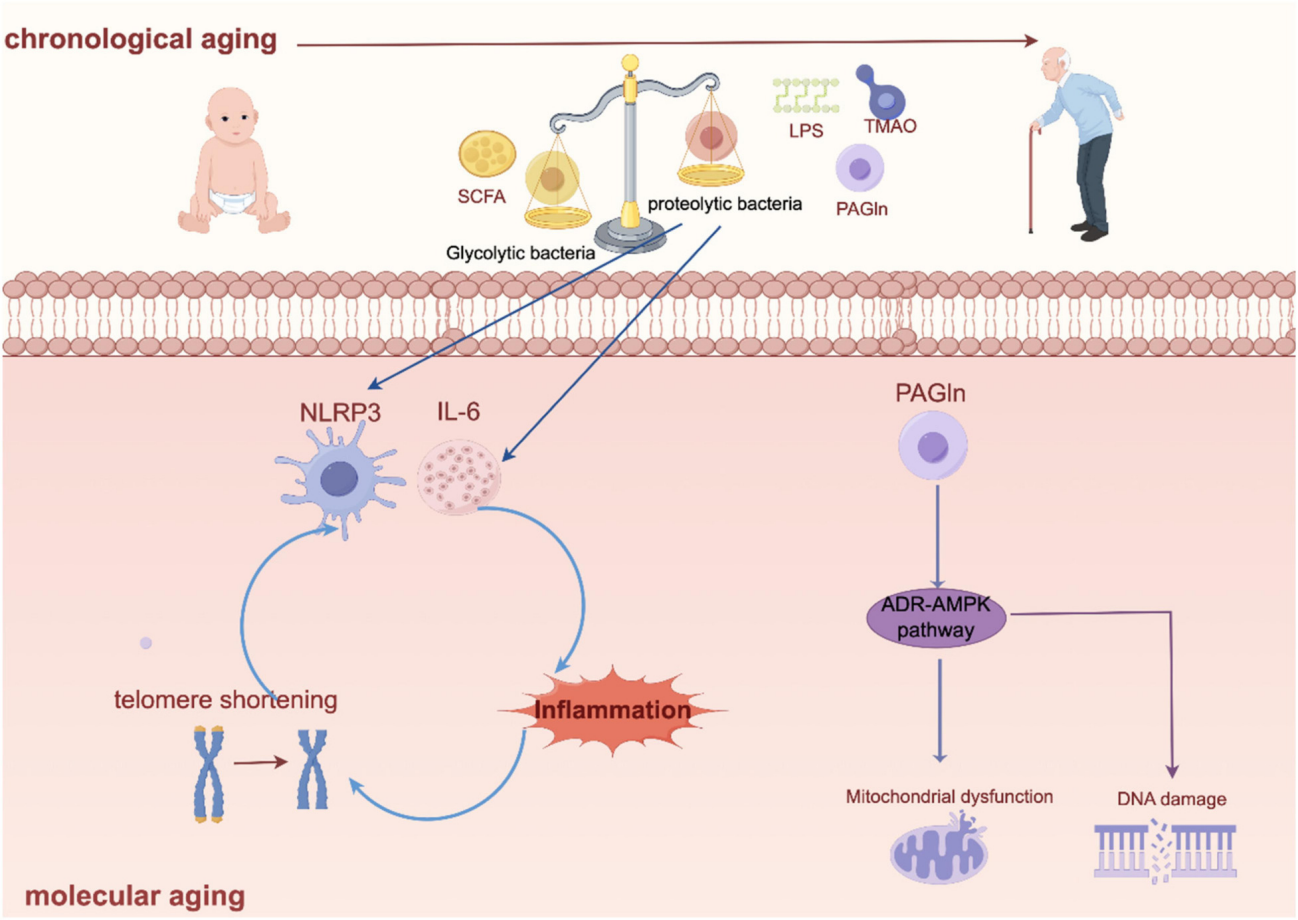
Intestinal flora leads to chronological ageing and molecular ageing. Created using Figdraw (https://www.figdraw.com/).

These findings not only confirm the strong association between age and gut flora, but also provide a theoretical basis for the development of microbiota intervention strategies for age-related diseases such as AF. Based on this evidence, modulation of the gut flora may be a new strategy for the prevention and treatment of cardiovascular disease in adults, which is expected to reduce the probability of cardiovascular events in the elderly population (Figure 2).

## 4 Intervention strategies targeting intestinal flora

### 4.1 Dietary regulation

Dietary structure is one of the key factors influencing the composition and function of intestinal flora (Table 3). Dietary patterns characterised by high fat and sugar have been shown to lead to a decrease in the abundance of beneficial flora such as bifidobacteria in the gut, which in turn triggers dysregulation of lipid metabolism (88) and local inflammatory responses (88). Whereas high dietary fibre intake in the Mediterranean diet (MD) significantly increased the proportion of thick-walled phyla (89), thick-walled phylum is able to produce butyric acid with anti-inflammatory effects, which plays an important role in maintaining microbial homeostasis and reducing LPS production (90). In addition adherence to MD significantly increased the level of SCFAs in faeces (91), however, inadequate adherence to MD can lead to elevated levels of TMAO in the blood, which can increase the risk of a number of chronic diseases, including cardiovascular disease (92). Dietary fibre also exerts its cardioprotective effects by modulating the renin-angiotensin-aldosterone (RAAS) system and blood pressure-related signalling pathways via SCFAs (89). Under the influence of a high-fibre diet, SCFAs and their metabolites interacted with the RAAS system in the host kidney and down-regulated RAAS in the kidneys of experimental rats, resulting in a substantial down-regulation of systolic and diastolic blood pressures (55). Animal studies confirm that a low dietary fibre diet leads to increased expression of Clostridium difficile, which in turn promotes the development of hypertension and cardiac remodelling (93). Treatment with SCFAs improves cardiovascular function through multiple mechanisms, including upregulation of intestinal tight junction protein mRNA expression levels, increased splenic Treg cell abundance, inhibition of pro-inflammatory factors (IL-17a, IL-6) and fibrosis markers (TNF-α, Col3a) expression, and mediation of G-protein-coupled receptor signal transduction (94). Collectively, these findings suggest that modifying dietary patterns may reduce cardiovascular risk through mechanisms such as promoting the production of SCFAs and attenuating the inflammatory response.

**Table 3:** Main evidence on dietary interventions targeting intestinal Flora.

Dietary pattern	Key impacts on gut flora & metabolites	Potential impact on AF risk and mechanisms
Mediterranean Diet (MD)	1. Significantly increases Firmicutes, particularly butyrate-producing bacteria.	Likely Reduces Risk Mechanisms: Butyrate has anti-inflammatory effects, maintains microbial homeostasis, and reduces LPS production. High fiber content modulates the RAAS system and blood pressure-related signaling pathways via SCFAs, lowering blood pressure (a major AF risk factor). Reduces pro-atherosclerotic and pro-thrombotic TMAO levels.
	2. Increases fecal SCFAs levels.	
	3. High adherence can reduce blood TMAO levels.	
High-Fiber Diet	1. Promotes the proliferation of SCFA-producing bacteria (e.g., Roseburia).	Mechanisms:
		1. SCFAs improve cardiovascular function via GPCR signaling.
	2. Significantly increases production of SCFAs (especially butyrate, propionate).	2. Upregulates intestinal tight junction proteins, enhancing the gut barrier and reducing endotoxemia.
		3. Inhibits pro-inflammatory factorsand fibrosis markers
	3. Low-fiber diet increases abundance of harmful bacteria like C. difficile.	4. Increases splenic Treg cell abundance, modulating immune response.
		5. Low-fiber diet promotes hypertension and cardiac remodeling.
High-Fat/High-Sugar Diet (Western Pattern)	1. Leads to a decrease in beneficial bacteria (e.g., Bifidobacteria).	Mechanisms:
		1. Triggers lipid metabolism disorders and local inflammatory responses.
	2. Creates favorable conditions for Gram-negative bacteria, increasing LPS (endotoxin) levels.	2. Compromises gut barrier function, allowing LPS into circulation and causing systemic chronic inflammation.
	3. Impairs colonic epithelial mitochondrial bioenergetics, promotes intestinal TMA production, and increases circulating TMAO levels.	3. High TMAO levels directly promote AF progression through inflammation, thrombosis, and fibrosis mechanisms.

SCFAs, short-chain fatty acids TMAO, trimethylamine N-oxide LPS, lipopolysaccharide RAAS, renin-angiotensin-aldosterone system; Treg, regulatory T cells.

While these beneficial mechanistic changes are corroborated by clinical outcomes (95, 96), extensive research has established the Mediterranean Diet as an effective strategy for cardiovascular disease prevention, demonstrating significant reductions in major adverse cardiovascular events, including myocardial infarction, stroke, and cardiovascular mortality. However, this clinical profile is nuanced: notably, a significant reduction in all-cause mortality has not been consistently verified across all studies (97). Furthermore, the limited reporting of adverse events in most research challenges a comprehensive assessment of the risk-benefit balance associated with the MD.

### 4.2 Supplementation of specific strains

Dai et al. found that six bacterial taxa- Fusicatenibacter, Holdemania, Howardella, Intestinibacter, Ruminococcaceae UCG014 and Turicibacter- are associated with a reduced risk of AF (98). Zuo et al. also found that (99) psaf-rich Holosporaceae family and Holospora genus, Methylovulum genus and Methylovulum miyakonense species were positively correlated with psaf-rich serum choline, which is the main factor contributing to atrial enlargement in AF patients. Study shows (100) the MD diet significantly increased the abundance of Clostridium difficile groups XVIa and Clostridium pumilus, which are not only involved in butyric acid and secondary bile acid production, but also play an important role in the formation of T-regulatory cells (101). Among them, butyric acid, as a histone deacetylase inhibitor, can exert significant anti-inflammatory effects by inhibiting the expression of pro-inflammatory cytokines (102). Furthermore, Roseburia species (103) also as one of the main producers of butyrate, which is negatively associated with cardiovascular complications associated with chronic kidney disease (CKD) (104). In summary, targeted supplementation of specific beneficial flora may also be a new strategy for the prevention of cardiovascular disease.

### 4.3 Exercise may play a role in preventing AF through gut flora

Numerous studies have shown that regular exercise is effective in improving intestinal barrier function and positively affecting the composition of intestinal flora (63). However, there were significant differences in the effects of different types of exercise on the gut flora (105).

Endurance exercise has been found to significantly alter the gut metabolite profile, as evidenced by increased levels of organic acids and decreased levels of nucleic acids. Notably, the effect of exercise on the metabolism of short-chain fatty acids (SCFAs) was weight-dependent: in lean individuals, running exercise significantly increased faecal SCFAs concentrations, whereas similar changes were not observed in obese individuals. This difference may be related to exercise-induced changes in the expression of the butyrate-regulated gene BCoAT and the propionate-regulated gene mmDA. Skeletal muscle-dominated exercise can potentially influence the composition of the gut flora, and it has now been found that exercise exerts beneficial regulatory effects on the composition and metabolic function of the gut flora (68). For example (106), the abundance of microorganisms involved in processes such as carbohydrate and amino acid metabolism and the production of SCFAs increased significantly, while playing a regulatory role in iron metabolism. From the point of view of the intestinal flora, this may be related to the elevated intestinal permeability which promotes the entry of pathogenic bacteria, lipopolysaccharides and other toxic substances into the body circulation, thus disrupting the internal homeostasis of the organism.

These findings provide new mechanistic explanations for the prevention of cardiovascular disease by exercise, and also suggest that gut flora may be an important target for exercise intervention (Table 4).

**Table 4:** Main evidence on supplementation with specific strains and exercise.

Intervention category	Specific intervention/finding	Impact on gut flora & key mechanisms	Potential effect on AF risk
Specific bacterial strains	Fusicatenibacter, Holdemania, Howardella, Intestinibacter, Ruminococcaceae UCG014, Turicibacter	Identification of these six bacterial taxa as being associated with a reduced risk of AF in Mendelian randomization analysis.	Likely Protective Mechanism: Their presence in the gut microbiome is causally linked to a lower incidence of AF, suggesting potential therapeutic benefits.
	Clostridium groups XVIa and Clostridium pumilus (enriched by MD diet)	1. Involved in butyrate and secondary bile acid production.	Likely Protective
		2. Play a crucial role in the formation of regulatory T cells (Tregs).	Mechanism: Butyrate acts as an HDAC inhibitor, exerting anti-inflammatory effects; Tregs modulate immune response and reduce inflammation.
	Roseburia species	One of the main producers of butyrate. Its abundance is negatively associated with cardiovascular complications in chronic kidney disease (CKD).	Likely Protective
			Mechanism: Butyrate production enhances intestinal barrier function, reduces systemic inflammation, and improves metabolic health.
	Holosporaceae family, Methylovulum genus	Positively correlated with serum choline, a major dietary precursor for the pro-arrhythmic metabolite TMAO. Associated with atrial enlargement in AF patients.	May Increase Risk
			Mechanism: These taxa may promote TMAO production, which facilitates AF through inflammation, fibrosis, and thrombosis.
Exercise	Endurance Exercise	1. Alters the gut metabolite profile (increases organic acids, decreases nucleic acids). 2. Effect on SCFA production is weight-dependent: increases fecal SCFAs in lean individuals but not in obese individuals. 3. Increases abundance of microbes involved in carbohydrate/amino acid metabolism and SCFA production.	Likely Protective
Mechanism: Improves intestinal barrier function, reduces permeability, and decreases entry of pathogens/LPS. Modulates iron metabolism. Promotes an anti-inflammatory environment.			
	Skeletal Muscle-Dominated Exercise	Exerts beneficial regulatory effects on the composition and metabolic function of the gut flora.	Likely Protective Mechanism: The gut-muscle axis may influence host physiology, potentially reducing systemic inflammation and improving metabolic parameters linked to AF risk.

MD, mediterranean diet; TMAO, trimethylamine N-oxide; SCFAs: short-chain fatty acids; Tregs, regulatory T cells; HDAC, histone deacetylase; LPS, lipopolysaccharide.

## 5 Evidence quality, disputes and challenges

This review systematically presents the role of the gut flora and its metabolites in AF. However, before translating these promising findings into clinical practice, it is imperative to critically evaluate the level of current evidence, existing controversies, and potential confounding factors.

The current body of evidence supporting the gut flora-AF axis primarily consists of animal studies and related observational research. Although animal models, such as the various rat and mouse models cited in this paper, have provided key evidence for elucidating the molecular mechanisms of metabolites like TMAO, PAGln, and LPS, significant interspecies differences must be acknowledged. For instance, mice and humans differ in both macroscopic and microscopic intestinal structures (107), as well as in cellular composition and function (108, 109). Furthermore, approximately 85% of mouse gut bacterial species are not present in the human gut (110). These fundamental differences highlight the need for a cautious evaluation of the relative importance and translatability of the relevant pathways in humans. For instance, while TMAO promotes AF in rodent models through pathways such as NLRP3 inflammasome activation, its association with AF in human observational studies has been inconsistent across different cohorts. Moreover, this association is often strongly confounded by renal function (111). Specifically, patients with reduced glomerular filtration rates (112) and those with chronic kidney disease exhibiting progressive renal impairment often exhibit elevated plasma TMAO levels (113). In contrast, TMAO levels can normalize following kidney transplantation (113), suggesting that the association between TMAO and AF may be partly driven by renal function status. Similarly, the pathogenic mechanisms of PAGln are currently supported mainly by cellular and animal experimental evidence (7, 85, 114). Its predictive value in broader populations and its causal relationship with AF require further validation through larger-scale prospective cohort studies and genetic evidence.

A core challenge in elucidating the relationship between gut flora and AF lies in disentangling the pervasive confounding factors. AF patients often present with multiple comorbidities, such as heart failure (115), hypertension (116), diabetes (117), and chronic kidney disease (118), and are frequently on long-term medications, including antibiotics, proton pump inhibitors, and antiarrhythmic drugs. These comorbidities and medications themselves are known to significantly alter the structure and function of the gut flora (119, 120). Consequently, it remains difficult to definitively determine whether the “AF-associated microbial signatures” observed in studies are a cause, a consequence, or an epiphenomenon of AF. Furthermore, diet is the strongest environmental factor shaping the microbiota. Variations in geographical regions, cultural backgrounds, and individual dietary habits can lead to difficulties in replicating and generalizing study findings (121). Host genetic background may also simultaneously influence susceptibility to AF and the colonization of the gut flora (122), further adding to the complexity of the analysis.

Current intervention studies targeting the microbiota, such as probiotic supplementation and dietary modifications, not only show inconsistent results (123) but have also raised safety concerns. For instance, a meta-analysis of 8 randomized controlled trials (*n* = 270) concluded that probiotic supplementation, overall, had no significant effect on circulating TMAO levels compared to controls (124). This finding directly challenges the presumed pathway from probiotic-modulated microbiota to reduced production of pro-arrhythmic metabolites. While a subgroup analysis suggested a potential benefit in individuals under 50 years of age, the primary null result underscores the gap between theoretical mechanisms and clinical outcomes. Cases of *Lactobacillus bacteremia* and complications have been reported in immunocompromised patients following long-term probiotic use (125). Regarding probiotics, although certain specific strains have shown antiarrhythmic potential in animal models, their effects often become weak or inconsistent when translated to human clinical trials. While the MD and dietary fiber demonstrate robust cardiovascular protective effects in observational studies, randomized controlled trial evidence regarding their direct impact on AF incidence remains limited (123, 126). These “unsuccessful” intervention studies are nonetheless of significant value, as they reveal the complexity of microbiota-targeted interventions and suggest that their efficacy may highly depend on the host's baseline microbiota, as well as the timing, dosage, and duration of the intervention. Future efforts will require more targeted and personalized intervention strategies.

## 6 Summary and outlook

In recent years, the role of gut flora in the development of AF has been gradually revealed. Gut flora and their metabolites are involved in the onset and development of AF through a variety of mechanisms, including inflammatory response, lipid metabolism, oxidative stress, atrial fibrosis, and regulation of the autonomic nervous system. These findings offer novel insights into AF pathogenesis and provide a theoretical basis for the development of targeted therapeutic strategies based on gut flora.

First, gut flora directly or indirectly promotes atrial inflammation and fibrosis through metabolites such as TMAO and LPS, increasing the risk of AF. Second, metabolites such as bile acids and short-chain fatty acids play an important role in regulating cardiac electrophysiological properties, inflammatory responses, and lipid metabolism, further influencing the onset and progression of AF. In addition, the interaction between gut flora and traditional risk factors for AF, such as hypertension, diabetes and hyperlipidemia, further emphasises the centrality of gut flora in AF. Based on these findings, gut flora and its metabolites are expected to be new targets for AF prediction, diagnosis and treatment. Future studies should further explore the causal relationship between gut flora and AF, and clarify the specific mechanism of action of specific flora and their metabolites in the development of AF. In addition, the development of intervention strategies based on gut flora (e.g., probiotics, faecal transplantation, etc.) may provide new directions for the prevention and treatment of AF.

Although a substantial body of research has revealed associations between gut flora and AF, establishing causality and translating these findings into effective therapies remain formidable challenges. Analysis of ongoing clinical trials (Table 5) reveals critical gaps that future studies must address with greater precision. Key unresolved questions include whether gut flora contributes equally to various AF subtypes (e.g., paroxysmal vs. persistent), and how demographic factors (age, gender, ethnicity) modulate this risk. To move beyond correlation, future research should prioritize three actionable strategies: First, employ humanized animal models—transplanting microbiota from well-phenotyped AF patients into germ-free mice—to directly test the arrhythmogenic potential of human microbial communities. Second, transition from generic probiotic trials to biomarker-enriched interventions, by recruiting patients with elevated pro-arrhythmic metabolites (e.g., TMAO, PAGln) to test targeted microbial therapies. Third, leverage metagenomic sequencing to identify not just correlated taxa, but the specific bacterial genes [e.g., the choline-utilizing *cutC/D* cluster (127)] responsible for producing AF-associated metabolites. By adopting these focused approaches, the field can advance from documenting associations to defining causal mechanisms and developing personalized interventions.

**Table 5:** Ongoing clinical trials investigating the role of Gut Flora in risk factors associated with AF.

Trial name	NCT or Identifier	Planned completion date	Study design & intervention	Primary outcome measures
Microbiota-Inflammation-Brain Axis in Heart Failure: New Functional Food for the Prevention of undeRnutrition in Older (AMBROSIA)	NCT07076329	30/08/2025	Interventional: Patients receiving probiotics vs. placebo after catheter ablation.	help improve muscle mass and physical performance in older adults with heart conditions
Impact of a probiotic supplement on menopause	ISRCTN56831337	31/12/2025	Interventional: All participants will receive a 12-week course of billions of live and active bacteria.	Questionnaire to measure quality of life
The survey of oral and intestinal microbiota in patients with diabetes and periodontitis and the effect of probiotic consumption	IRCT20250107064315N1	—	Interventional: One probiotic capsule daily for 12 weeks	Quantitative real-time_ Polymerase Change Reaction.
effects of synbiotics on protein absorption for muscle mass improvement in recreational male bodybuilders	TCTR20250803008	31/12/2025	Interventional: Placebo Comparator Dietary Supplement	Probiotic levels pre-test, post-test Colony forming units
comparison of zinc and probiotics on neonates with indirect hyperbilirubinemia undergoing phototherapy ZIPP	NCT07102836	—	interventional: Drug1: Zinc sulfate Drug2: Probiotic lactobacillus rhamnosus GG	Rate of decline in Bilirubin levels during phototherapy with this adjuvant therapy
Effect of probiotic on chemotherapy-induced side effects	IRCT20130211012438N37	—	Interventional: One probiotic capsule daily for 12 week	Chemotherapy induced side effects include gastrointestinal symptoms such as nausea
Adipose Tissue Gene Expression and Metabolomics Links to the Gut Microbiome-brain Axis POINSETTIA	NCT06869941	—	Observational: Blood sugar and percentage of deep sleep	Possible links between lipid metabolism and gut flora
How a Single Workout Affects Gut Bugs in Women With Different Fitness Levels and Body Types FITGut-W	NCT06691100	—	Interventional: aerobics	Gut Microbiome Composition: Before the exercise intervention and immediately after the intervention
